# Repeated Genicular Artery Embolization Using Permanent Microspheres for Severe Osteoarthritis and Postsurgical Pain

**DOI:** 10.1007/s00270-026-04410-w

**Published:** 2026-03-18

**Authors:** A. Taheri Amin, A. Hübner, L. Abu-Gharbieh, E. Kemmer, P. Krüselmann, F. Ziayee, L. M. Wilms, C. B. Fink, K. Jannusch, P. Minko

**Affiliations:** 1 Department of Diagnostic and Interventional Radiology, Charité Universitätsmedizin Berlin, Corporate Member of Freie Universität Berlin and Humboldt-Universität Zu Berlin, Campus Virchow-Klinikum (CVK), Augustenburger Platz 1, 13353 Berlin, Germany; 2 Department of Diagnostic and Interventional Radiology, Medical Faculty, University Hospital Duesseldorf, 40225 Duesseldorf, Germany

**Keywords:** Genicular Artery Embolization, GAE, Reperfusion in GAE, Repeat-GAE, Double GAE, Embolization, knee OA

## Abstract

**Purpose:**

To investigate reperfusion after genicular artery embolization (GAE) in patients with severe osteoarthritis (OA) or persistent pain after total knee replacement (post-TKR), who did not achieve clinical improvement after initial GAE, and to evaluate the clinical efficacy of repeat GAE (reGAE).

**Materials and Methods:**

This prospective observational study included patients with radiographically severe OA or post-TKR pain. GAE was performed using permanent microspheres. Clinical outcome was assessed at 6 weeks, 3, 6, 9, and 12 months using the numeric rating scale (NRS). Minimal clinically important difference (MCID) was defined as an improvement of at least 2 points compared with baseline. Patients failing to achieve MCID at 6 months underwent reGAE. Angiographic blush size before and after embolization during GAE and reGAE was measured and compared.

**Results:**

In 55 patients (87 GAEs), a median of 4 (range, 2–6) vessels was treated, with a median total embolic volume of 4.5 mL (1.5–10.1 mL). After initial GAE, 23 patients (42%) achieved MCID. Following reGAE at 6 months, an additional 20 patients (36%) reached MCID, with sustained efficacy up to 6 months after reGAE (*p* ≤ 0.0001); 12 patients (22%) remained non-responders. Quantitative angiographic analysis demonstrated a significant increase in blush size within previously treated vessels, necessitating reGAE (*p* ≤ 0.0001).

**Conclusion:**

After GAE using permanent microspheres, reperfusion of previously treated vessels was observed at 6 months in all patients failing to achieve MCID. ReGAE increased the proportion of clinical responders, supporting its role as an effective additive treatment strategy in severe OA and post-TKR pain.

**Graphical Abstract:**

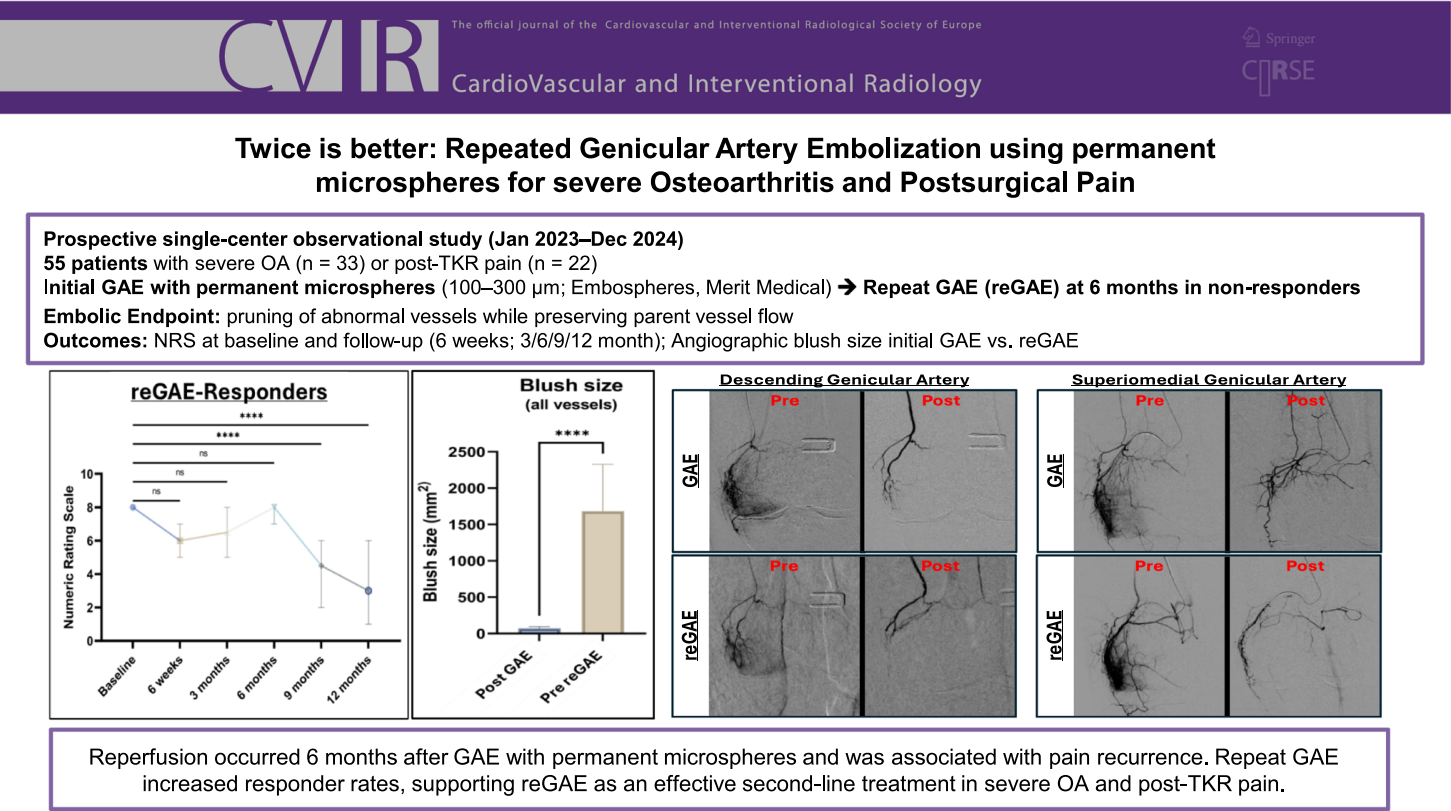

**Supplementary Information:**

The online version contains supplementary material available at 10.1007/s00270-026-04410-w.

## Introduction

Genicular artery embolization (GAE) is an effective treatment option for refractory knee pain in osteoarthritis (OA) with clinical efficacy demonstrated in several meta-analyses [1–3]. Despite rising procedural volumes, important questions regarding patient selection and procedural technique remain unresolved.

The efficacy of GAE in patients with severe OA and persistent pain after total knee replacement (post-TKR) has been insufficiently investigated, with heterogeneous results reported [4]. While previous work from our group demonstrated that higher volumes of permanent embolics and a higher number of treated vessels can achieve sustained clinical efficacy in severe OA, earlier studies using temporary embolics reported pain recurrence within 3 to 6 months [5–8].

Evidence from PET/CT studies for adhesive capsulitis suggests that while chronic inflammation can be reduced by embolization, it may persist even after treatment [9]. In GAE, these observations are further accentuated by technical and anatomical characteristics. Temporary embolics used in GAE typically undergo recanalization within hours, and even permanent embolics have been shown to fragment and undergo resorption over time, especially in inflamed tissue [10, 11]. Additionally, the knee joint is characterized by an extensive anastomotic network capable of collateralizing embolized territories [12]. Consequently, in addition to the chronic progressive nature of synovitis sustained efficacy of GAE may also be limited by recanalization of embolics and the extensive collateral network of the knee joint.

This study aimed to investigate whether reperfusion of previously treated vessels occurs after initial GAE with permanent embolics in patients with severe OA and post-TKR pain, who fail to achieve clinical success. In addition, we evaluated whether repeat GAE (reGAE) can increase the proportion of responders in this cohort.

## Materials and Methods

### Study Design

This prospective, single-center observational study was performed at the University Hospital of Duesseldorf between January 2023 and December 2024. Patients aged 18–90 years with radiographic evidence of severe knee OA or post-TKR were eligible if symptoms were refractory to at least six months of best medical therapy. In patients with bilateral knee OA, inclusion required that the predominant side of pain was concordant to the side with radiographically severe OA. Only the knee fulfilling these criteria was treated. Patients with relevant axial malalignment of the knee (varus or valgus deformity > 10°) and patients who underwent bilateral GAE were excluded from the study. Other exclusion criteria included pregnancy, severe peripheral arterial disease (Rutherford grade ≥ III), active or suspected knee infection, irreversible coagulopathy, bleeding disorders, and impaired renal function.

The study was approved by the institutional review board, and all procedures were performed in accordance with the Declaration of Helsinki. Written informed consent was obtained from all participants prior to inclusion. This study did not involve interventional assignment beyond standard clinical care and was therefore not registered in a public trial registry.

### Procedure

All procedures were performed by a single IR with more than 15 years of experience (P.M.) to minimize inter-operator variability. The IR was aware of the site of maximal pain prior to the procedure. GAE was performed via an ipsilateral antegrade transfemoral approach without an introducer sheath. DSA was performed at the mid-third of the distal superficial femoral artery using a 4F Cobra catheter (Infiniti, Cordis Medical, Austria) and iodinated contrast medium (300 mg/mL Accupaque, GE Healthcare, USA) to delineate vascular anatomy. Superselective catheterization of all visible genicular arteries was achieved with a 1.7F microcatheter (Pursue, Merit Medical, USA). Embolization was performed upon detection of a hyperemic blush with 100–300-µm permanent microspheres (Embospheres, Merit Medical, South Korea) diluted in 10 mL of the iodinated contrast medium mentioned above. Embolic material was administered in small aliquots to a subjective angiographic endpoint, defined as pruning of pathological neovessels while preserving antegrade flow in the parent vessel. ReGAE was performed using the same protocol as initial GAE, with selective catheterization of all genicular arteries visible on overview DSA and embolization to the endpoint of pruning when a vascular blush was identified.

Patients were observed for 4 h post-procedure in the outpatient clinic before discharge. Vascular complications were assessed clinically and by duplex/doppler ultrasound prior to discharge and at 24 h. All peri- and post-procedural complications were documented and classified according to the modified CIRSE Quality Assurance Guidelines and Standards for Complication Reporting [13].

Prior to the intervention, patients were asked to indicate the site of maximal knee pain (medial or lateral). Overall pain was assessed using the numeric rating scale (NRS) at baseline and at 6 weeks, 3, 6, 9, and 12 months post-intervention.

### Image Review

Pre-interventional radiographs were independently assessed by two musculoskeletal radiologists (L.W. and K.J.), and study inclusion was limited to patients with severe OA according to the Kellgren–Lawrence scale or post-TKR. DSA images of all procedures were independently reviewed by a radiology resident (E.K.) and a research associate (A.H.), both blinded to patient identity and procedural data. For each treated vessel, pre- and post-embolization series were selected. Images with motion artifacts or markedly different catheter positions between pre- and post-embolization series and between initial and reGAE were excluded.

Eligible DSA series were post-processed in ImageJ/FIJI (Laboratory for Optical and Computational Instrumentation, University of Wisconsin, USA) and converted into color-coded images (cc-DSA) as previously described [14]. Blush areas of cc-DSA images were segmented in 3D Slicer (Version 5.6.2) and quantified in mm^2^ [15]. All segmentations were reviewed by a second radiologist (A.T.), and discrepancies were resolved in consensus. For each vessel, the pre- and post-embolization blush sizes and the percentage change (blush reduction ratio; BRR) during initial and reGAE were recorded.

### Study Endpoints

The primary technical endpoint was demonstration of reperfusion. Reperfusion was defined as a significant increase in blush size prior to reGAE compared to the post-embolization blush size after initial GAE within the same vessel. The primary clinical endpoint was achievement of the minimal clinically important difference (MCID), defined as a reduction in NRS of ≥ 2 points [16], assessed 6 months after initial GAE and reGAE.

Secondary endpoints included technical success, defined as catheterization of all angiographically visible genicular arteries. Further secondary endpoints were comparisons of embolic volume, number of embolized vessels, baseline blush size, and BRR between initial GAE and reGAE, as well as between responders and non-responders. In addition, baseline blush size was compared between medial and lateral genicular arteries according to the site of maximal pain. Safety endpoints included the occurrence of skin discoloration or other vascular/non-vascular complications.

### Statistical Analysis

This observational study had an exploratory design. Thus, no sample size calculation was performed. The target sample was determined by consecutive recruitment during the study period.

Normality of variables was assessed using the Shapiro–Wilk test. Data are expressed as mean ± SD for normally distributed variables and as median (range) for non-normally distributed variables. All parameters were non-normally distributed. Intraindividual changes in NRS at each follow-up compared to baseline were analyzed using the Wilcoxon signed-rank test, with p values adjusted for multiple comparisons using the Holm–Šídák method.

Responder status was defined as achievement of MCID and assessed 6 months after initial GAE and 6 months after reGAE. Accordingly, subgroups were defined as initial GAE responders, reGAE responders and non-responders. Changes in responder status between the two time points were analyzed using exact McNemar’s test for paired binary data. Intergroup differences in embolic volume, number of embolized vessels, NRS, baseline blush size, and BRR were assessed using the Kruskal–Wallis test, followed by Dunn’s multiple comparisons test. To evaluate reperfusion, post-embolization blush size after initial GAE was compared with baseline blush size prior to reGAE in patients undergoing reGAE, using the Wilcoxon signed-rank test.

Patients were further stratified according to the site of maximal pain (medial vs. lateral). Baseline blush size in medial and lateral genicular arteries was compared using the Wilcoxon signed-rank test.

Statistical significance was set at p < 0.05. Statistical analyses were performed using Microsoft Excel (Excel 2024) and GraphPad Prism (Prism 10.6.0).

## Results

A total of 55 patients were included in this study. Patient characteristics are summarized in Table 1. Technical success was achieved in all patients during both initial and reGAE (Table 2). No patients were lost to follow-up.

**Table 1:** Patient characteristics

	Total (n = 55)	GAE Responders (n = 23)	reGAE Responders (n = 20)	Non-Responders (n = 12)
Age (years) median (range)	69 (42–94)	69 (48–94)	72 (42–87)	66 (52–84)
Female. n (%)	32 (58%)	16 (70%)	11 (55%)	5 (42%)
BMI. median (range)	25 (18–42)	23 (18–37)	27 (20–38)	28 (20–42)
Pain localization n (medial:lateral)	38:17	15:8	15:5	8:4
Side n (%)				
Right	34 (62%)	12 (52%)	14 (70%)	8 (67%)
Left	21 (38%)	11 (48%)	6 (30%)	4 (33%)
OA severity				
K&L 4	33 (60%)	17 (74%)	10 (50%)	6 (50%)
Post-TKR	22 (40%)	6 (26%)	10 (50%)	6 (50%)

Abbreviations: BMI: body mass index, OA: osteoarthritis, K&L: Kellgren and Lawrence Scale; Post-TKR: post-total knee replacement

**Table 2:** Interventional data

	Total (n = 87)	GAE (n = 55)	reGAE (n = 32)
Fluoroscopy time (minutes). median (range)	26 (4–62)	26 (7–57)	23 (4–62)
Cumulative air kerma (mGy). median (range)	120 (9–488)	110 (10–387)	99 (9–488)
*Arteries embolized n (%)*			
Total	312	193	119
DGA	58 (67%)	35 (64%)	23 (72%)
SMGA	44 (51%)	28 (51%)	16 (50%)
IMGA	65 (75%)	39 (71%)	26 (81%)
SLGA	64 (74%)	41 (48%)	23 (72%)
ILGA	73 (61%)	45 (82%)	28 (88%)
ARTA	8 (9%)	5 (9%)	3 (9%)
*Number of arteries embolized*			
median (range)	4 (2–6)	4 (2–5)	4 (2–6)
2 n (%)	4 (5%)	2 (4%)	2 (6%)
3 n (%)	35 (40%)	25 (46%)	10 (31%)
4 n (%)	42 (48%)	26 (47%)	16 (50%)
5 n (%)	5 (6%)	2 (4%)	3 (9%)
6 n (%)	1 (1%)	0	1 (3%)
*Embolic volume (mL)* median (range)			
Total	4.5 (1.5–10.1)	4.0 (1.1–10.1)	4.7 (2.1–9.3)
DGA	1.8 (0.5–3.0)	1.8 (0.5–3.0)	1.6 (0.8–3.0)
SMGA	1.3 (0.3–5.0)	1.3 (0.3–5.0)	1.2 (0.5–5.0)
IMGA	1.0 (0.3–2.5)	1.0 (0.3–2.4)	1.0 (0.3–2.5)
SLGA	1.0 (0.3–3.5)	1.0 (0.3–3.5)	1.0 (0.5–3.5)
ILGA	1.0 (0.3–4.0)	0.9 (0.3–2.5)	1.2 (0.4–4.0)
ARTA	1.1 (0.6–2.0)	1.0 (0.6–1.5)	1.6 (0.8–2.0)
*Segmented arteries n (%)*			
Total	239	150 (78%)	89 (75%)
DGA	54 (93%)	33 (94%)	21 (91%)
SMGA	29 (66%)	18 (64%)	11 (69%)
IMGA	54 (83%)	32 (82%)	22 (85%)
SLGA	37 (58%)	25 (61%)	12 (52%)
ILGA	58 (80%)	37 (82%)	21 (75%)
ARTA	7 (88%)	5 (100%)	2 (67%)

Abbreviations: DGA: descending genicular artery, SMGA: superomedial genicular artery, IMGA: inferomedial genicular artery, SLGA: superolateral genicular artery, ILGA: inferolateral genicular artery, ARTA: anterior recurrent tibial artery

**Fig. 1:**
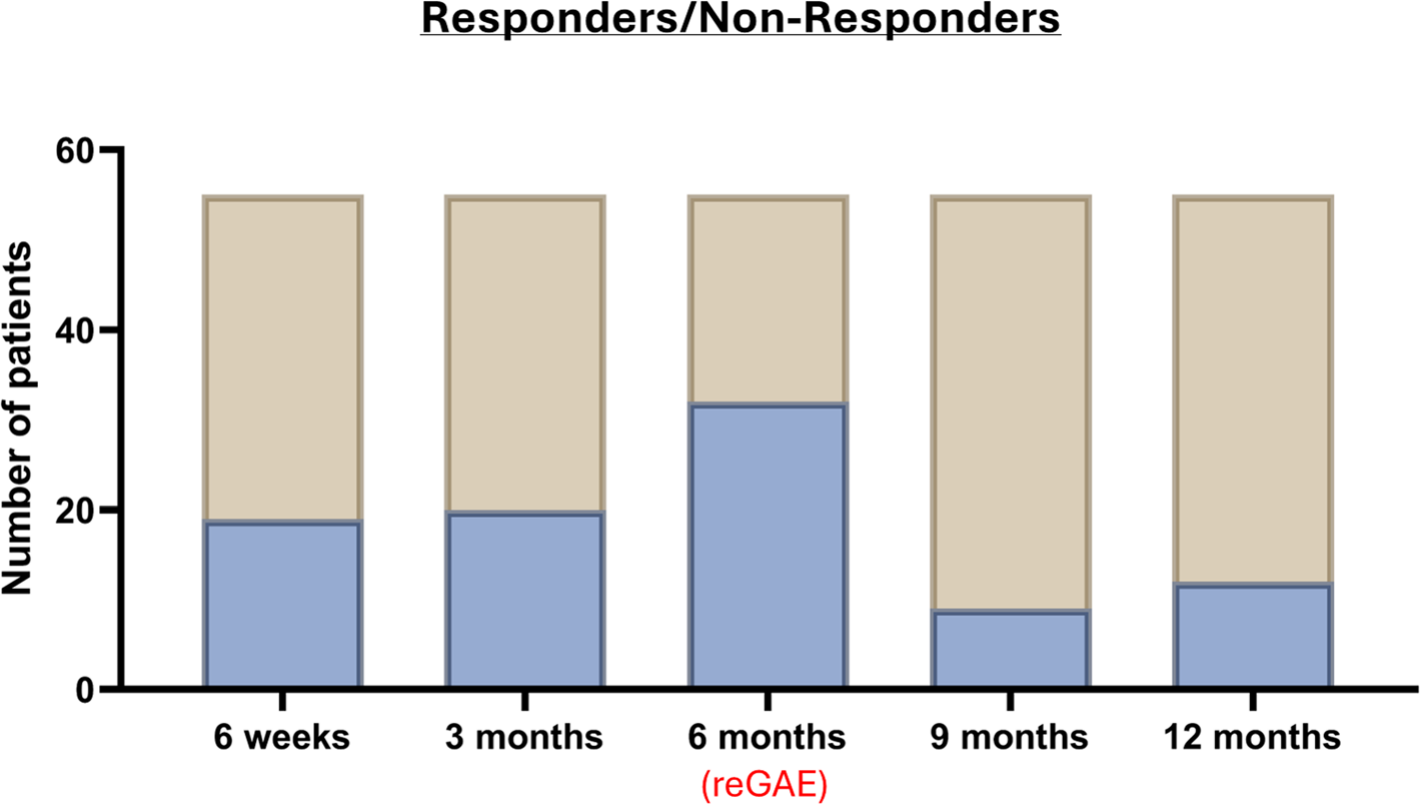
Responder rate after repeat GAE. Stacked bar chart showing the number of responders (beige) and non-responders (blue) at 6 weeks and at 3, 6, 9, and 12 months after GAE. Response was defined as achieving a minimal clinically important difference (MCID) of ≥ 2-point reduction in numeric rating scale compared with baseline. After 6 months, the proportion of responders increased following repeat GAE (reGAE in red)

**Fig. 2:**
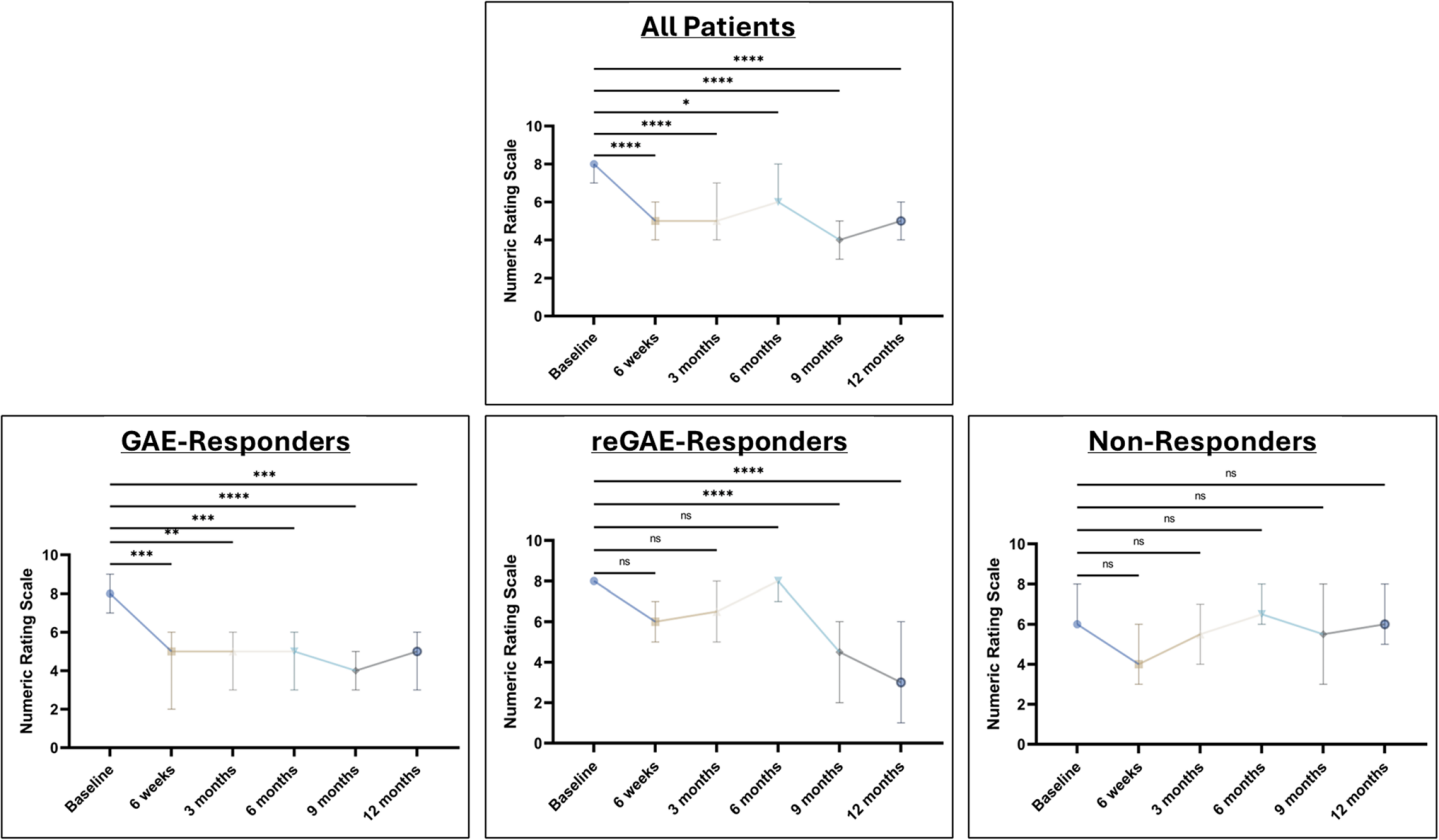
Outcome of initial and repeat GAE. Numeric rating scale (NRS) scores over time for all patients (top) and stratified into GAE responders, repeat GAE (reGAE) responders, and non-responders (bottom). NRS was assessed at baseline, 6 weeks and 3, 6, 9, and 12 months. Patients not achieving a minimal clinically important difference (MCID; ≥ 2-point NRS reduction from baseline) at 6 months underwent repeat GAE. Data are shown as median and range. Abbreviations: *p ≤ 0.05; **p ≤ 0.01; ***p ≤ 0.001; ****p ≤ *0.0001; ns, not significant*

At 6 months after initial GAE, 23 patients (42%) reached MCID and were classified as responders. Among the 32 initial non-responders, 20 patients (63%) achieved MCID 6 months after reGAE. Overall, 43 of 55 patients (78%) were classified as responders at 12 months (Figs. 1 and 2; Supplement 1). McNemar’s exact test demonstrated a significant increase in responder status between 6 and 12 months (*p* ≤ 0.001) (Supplement 2).

**Fig. 3:**
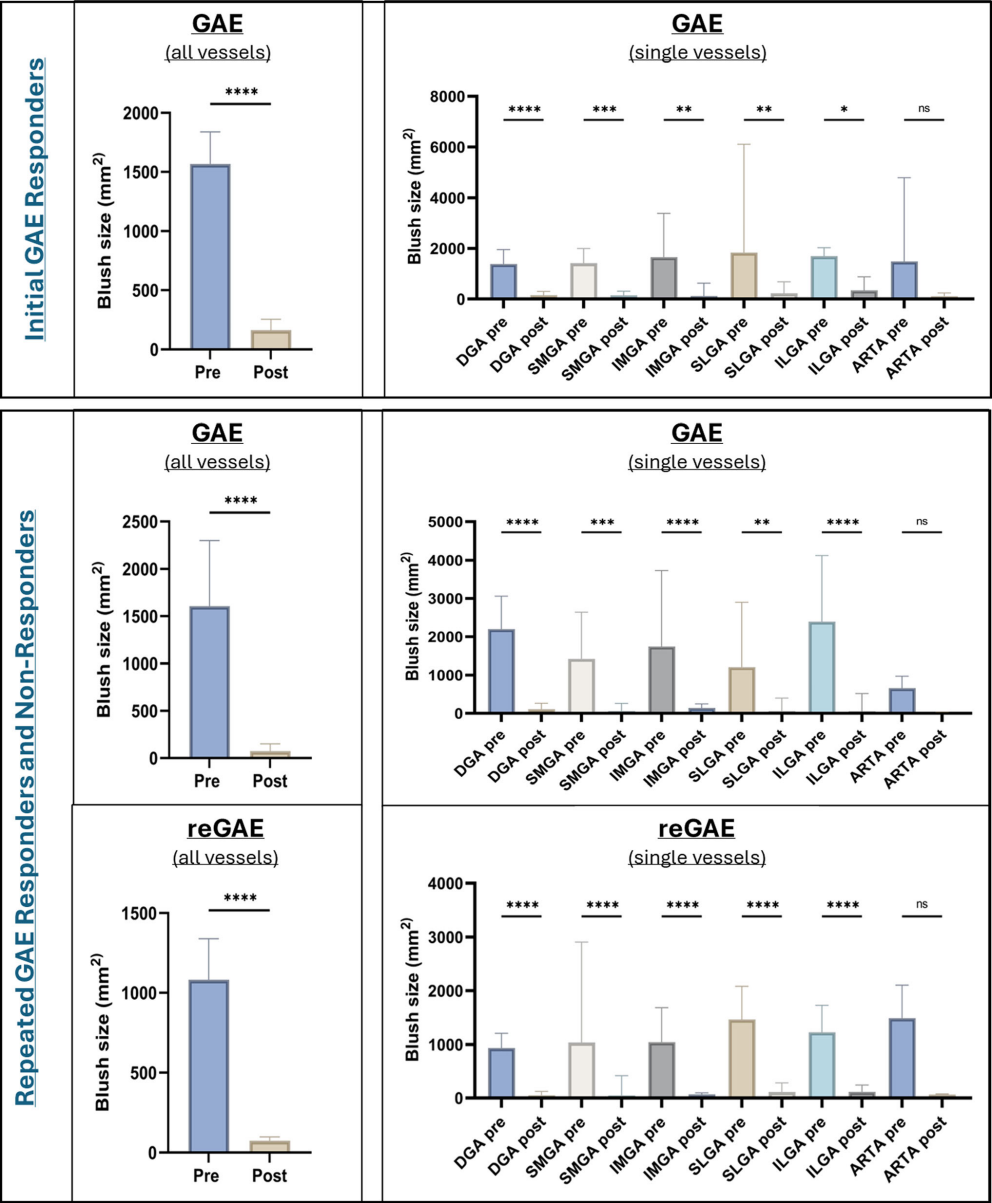
Changes of blush size after initial and repeated GAE. Bar charts depict angiographic blush size before and after embolization during initial genicular artery embolization (GAE) and repeat GAE (reGAE). Data are shown for all treated vessels (left panels) and stratified by individual genicular arteries (right panels). The upper panels show blush size changes after initial GAE in initial GAE responders, defined as patients achieving the minimal clinically important difference (MCID; ≥ 2-point reduction in NRS) within 6 months after the initial procedure. The middle panels display blush size changes after initial GAE in patients who did not achieve MCID at 6 months and subsequently underwent reGAE. This cohort includes both reGAE responders and non-responders. ReGAE responders were defined as patients achieving MCID within 6 months after reGAE, whereas non-responders failed to achieve MCID despite repeat intervention. The lower panels show blush size changes after reGAE in the same patient cohort. A significant reduction in blush size was observed after embolization during both initial GAE and reGAE across all treated vessels and in single-vessel analyses, with the exception of the anterior recurrent tibial artery, indicating effective devascularization. Abbreviations: *p ≤ 0.05; **p ≤ 0.01; ***p ≤ 0.001; ****p ≤ *0.0001; ns, not significant; DGA: descending genicular artery; SMGA: superiomedial genicular artery; IMGA: inferiomedial genicular artery; SLGA: superolateral genicular artery; ILGA: inferiolateral genicular artery; ARTA: anterior recurrent tibial artery*

In both subgroups of initial GAE responders (Supplement 3) and the groups of reGAE responders and non-responders (Supplement 4), embolization resulted in a significant reduction in blush size in treated vessels, both collectively and when stratified by individual genicular arteries, with the exception of the anterior recurrent tibial artery (ARTA) (Fig. 3). When stratified by responder status, baseline NRS, number of embolized vessels, total embolic volume, baseline blush size, and BRR did not differ significantly between responders and non-responders after initial GAE or reGAE.

No significant differences in blush size between medial and lateral genicular arteries were observed in patients with medial or lateral sites of maximal pain.

**Fig. 4:**
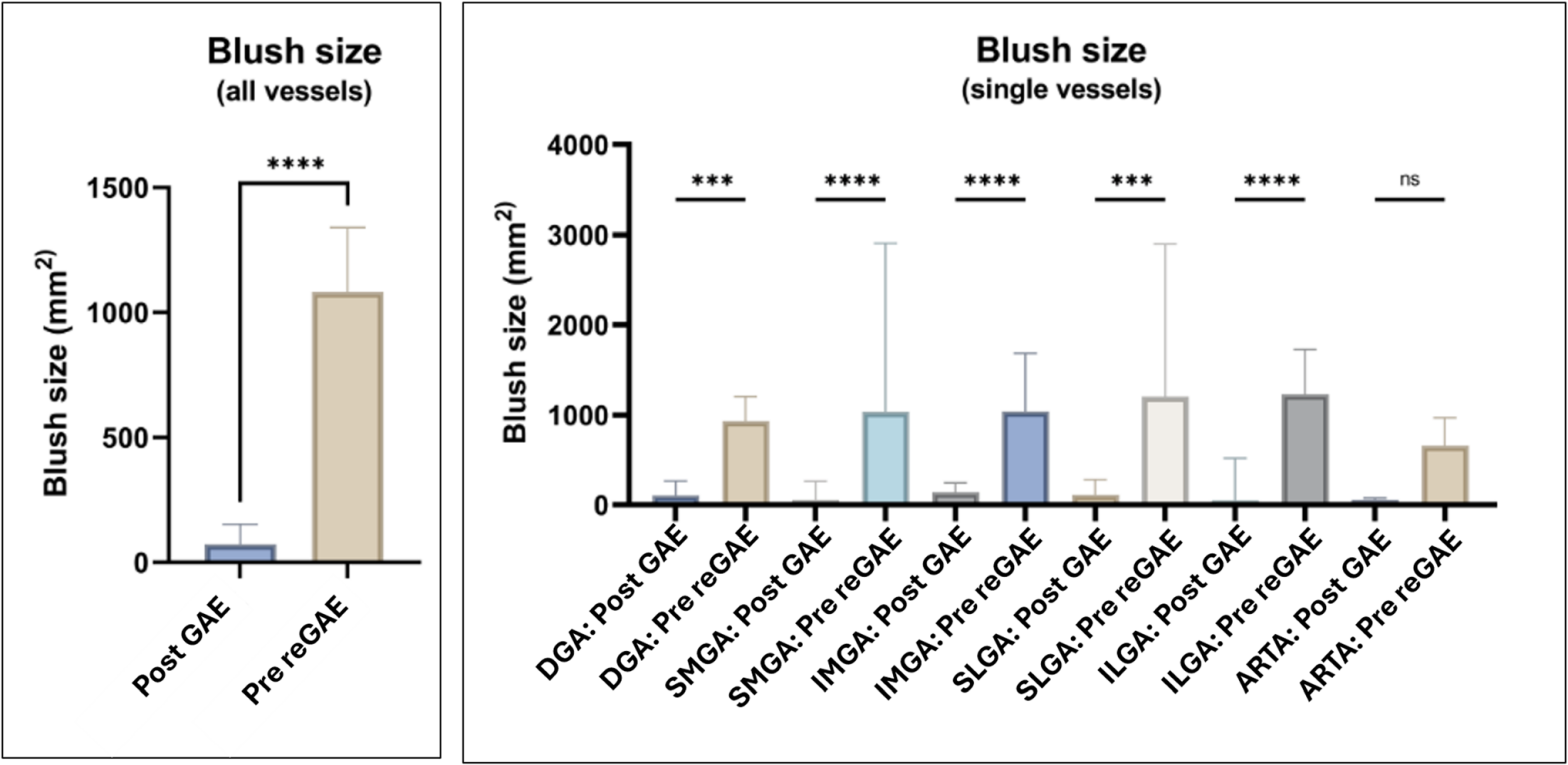
Reperfusion in Repeat GAE. Bar charts show comparing angiographic blush size post-embolization after initial GAE with pre-embolization before repeat GAE (reGAE), displayed for all vessels and stratified by individual genicular arteries. A significant increase in blush size between post-GAE and pre-reGAE measurements indicates reperfusion of previously embolized vascular territories, necessitating repeat embolization. Abbreviations: *p ≤ 0.05; **p ≤ 0.01; ***p ≤ 0.001; ****p ≤ *0.0001; ns, not significant; DGA: descending genicular artery; SMGA: superiomedial genicular artery; IMGA: inferiomedial genicular artery; SLGA: superolateral genicular artery; ILGA: inferiolateral genicular artery; ARTA: anterior recurrent tibial artery*

**Fig. 5:**
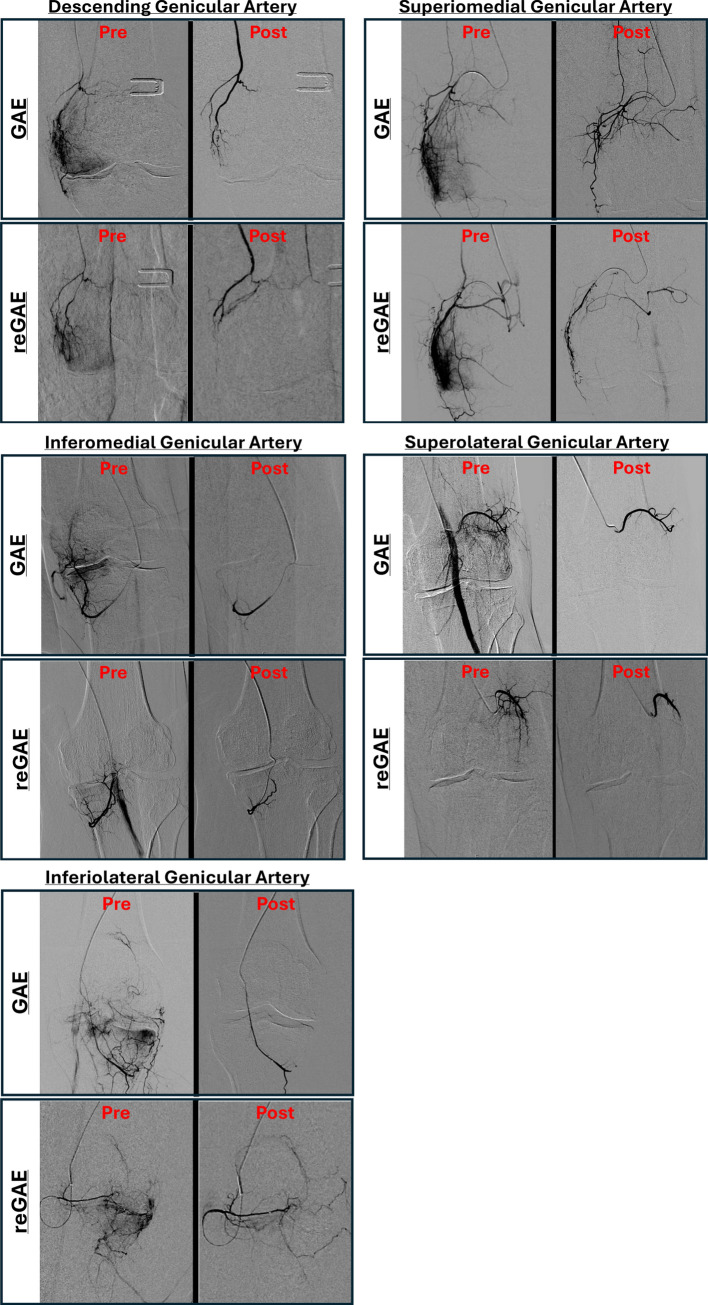
Reperfusion in repeat GAE. Representative DSA images demonstrating reperfusion in previously embolized genicular arteries. For each vessel, pre-embolization (Pre) and post-embolization (Post) angiograms from the initial genicular artery embolization (GAE) and from repeat GAE (reGAE) are shown. Images depict five different genicular arteries from five different patients (one representative example per artery). During the initial GAE, embolization was performed using 100–300 µm permanent microspheres, resulting in a marked reduction in angiographic blush with adequate pruning of pathological neovessels. At the time of reGAE, angiography demonstrated recurrent blush within the same vascular territories, consistent with reperfusion and necessitating repeat embolization. These examples illustrate that reperfusion of previously treated genicular arteries can occur despite technically successful embolization with permanent embolics. Note: Due to variable DSA acquisition lengths, phase matching between pre- and post-embolization images was not consistently feasible. For quantitative analysis, all frames of each DSA series were fused to account for phase variability

**Fig. 6:**
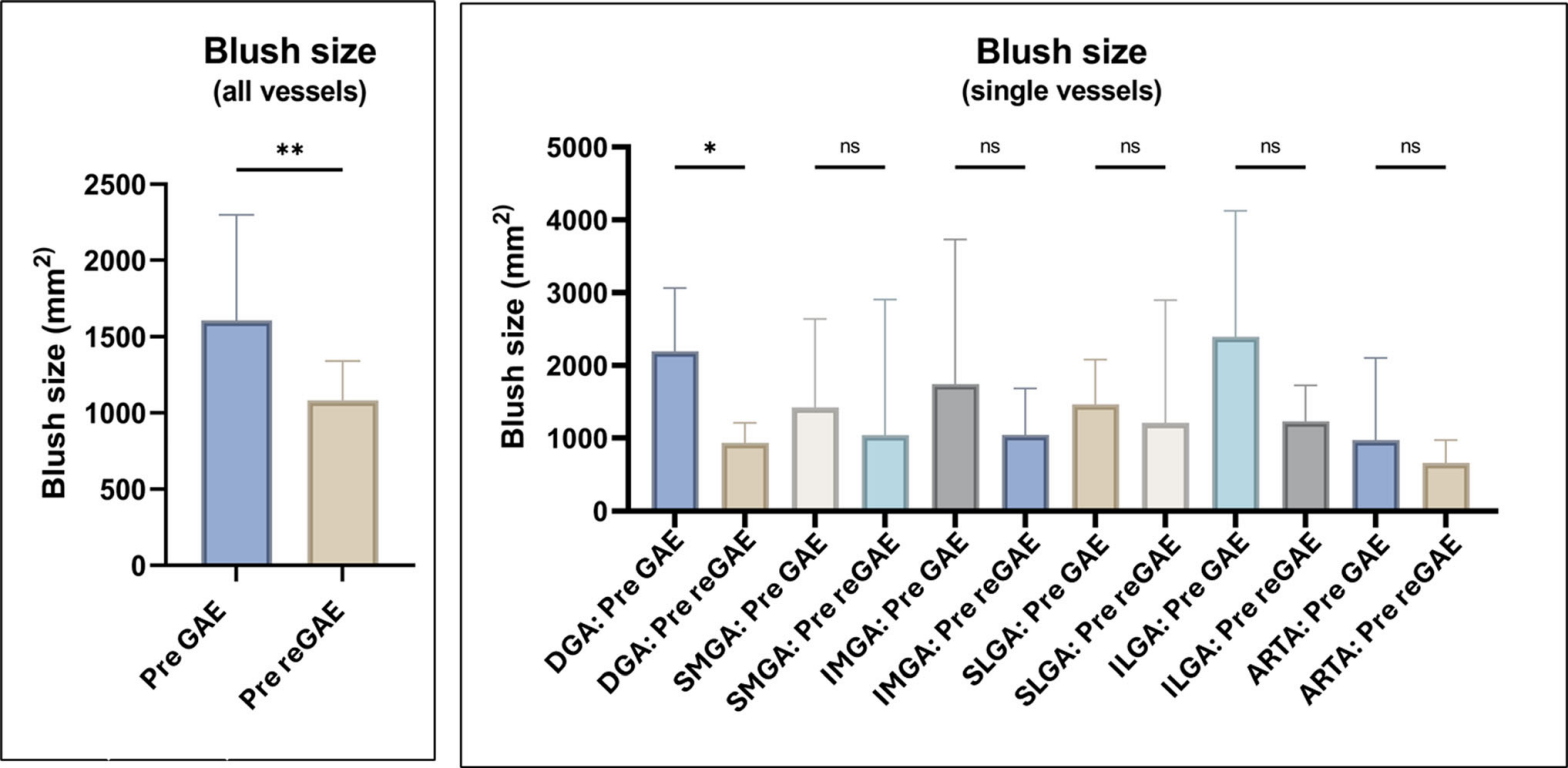
Baseline Blush size in initial and repeat GAE. Bar charts depict baseline blush size measured before embolization at the time of the initial GAE and before repeat GAE (reGAE). Data are shown collectively for all treated vessels (left panel) and stratified by individual genicular arteries (right panel). When analyzed across all vessels, baseline blush size prior to initial GAE was significantly greater than baseline blush size prior to reGAE. On vessel-level analysis, this difference reached statistical significance only for the descending genicular artery (DGA), while no significant differences were observed for the remaining genicular arteries. Abbreviations: *p ≤ 0.05; **p ≤ 0.01; ***p ≤ 0.001; ****p ≤ *0.0001; ns, not significant; DGA: descending genicular artery; SMGA: superiomedial genicular artery; IMGA: inferiomedial genicular artery; SLGA: superolateral genicular artery; ILGA: inferiolateral genicular artery; ARTA: anterior recurrent tibial artery*

In patients undergoing reGAE due to lack of clinical improvement at 6 months, baseline blush size prior to reGAE was significantly greater than post-embolization blush size after initial GAE (Figs. 4, 5; Supplement 4). When stratified by individual genicular arteries, this increase was likewise significant for all vessels except the ARTA. Baseline blush size prior to initial GAE was significantly greater than baseline blush size prior to reGAE when analyzed collectively across all vessels. When stratified by individual genicular arteries, this difference was significant only for the descending genicular artery (Fig. 6; Supplement 4). Transient skin discolorations occurred in 26 (47%) patients after initial GAE and in 13 (41%) patients after reGAE. All cases resolved completely by the six-week follow-up. No other vascular or non-vascular complications were observed.

## Discussion

This study observed that in patients with severe OA and post-TKR pain, who show insufficient initial response to GAE, reperfusion of previously treated vessels can occur despite the use of permanent embolics. ReGAE in these patients was associated with an increase in the proportion of responders.

Only a limited number of studies have investigated the outcomes of GAE in advanced OA and post-TKR pain, reporting heterogeneous results [6–8, 17]. In studies using temporary embolic agents that failed to demonstrate sustained efficacy, a pattern similar to our cohort was observed, with initial pain improvement at 6 weeks followed by pain recurrence at 3- and 6-month follow-up [7].

Okuno et al. demonstrated that in patients with mild to moderate OA, reGAE performed after four months resulted in an increased clinical success rate [18]. Our study extends these findings to patients with severe OA and post-TKR pain.

Repeat intervention was associated with a statistically significant increase in the number of patients achieving MCID, with clinical benefit persisting for up to six months after reGAE. The number of embolized vessels, embolic volume and BRR did not differ significantly between the initial GAE and reGAE, indicating consistent technique. However, within the same vascular territories, blush size prior to reGAE was significantly greater than post-embolization blush size after initial GAE. Thus, despite the use of permanent embolics and consistent embolization technique, patients undergoing reGAE due to lack of clinical improvement showed reperfusion of previously treated vascular territories within six months. Reperfusion may therefore represent a potential mechanism underlying persistent pain after initial GAE.

This observation is supported by technical aspects of GAE and the underlying pathophysiology of OA pain. OA pain is mainly driven by low-grade synovitis, which is chronically progressive and although reduced by transarterial embolization may persist over time [9, 19]. Temporary embolics used in GAE typically undergo recanalization within days, and even permanent embolics have been shown to recanalize within weeks, particularly in inflamed tissue as encountered in OA [10, 11]. Recanalization may be further facilitated by the more restrictive embolization endpoint of pruning when using permanent embolics [1, 20]. However, even with more aggressive embolization endpoints using temporary embolics—such as stasis/reflux during GAE with Lipiodol—washout of Lipiodol over time is well described [21]. In addition, the knee joint is characterized by an extensive anastomotic network that may compensate for embolized vessels through collateralization, thereby limiting the embolic effect [22]. In prostate artery embolization (PAE), reperfusion of previously treated vessels and the resulting need for repeat PAE are well recognized [23, 24]. Our study translates this concept to GAE by linking procedural technique with the pathophysiology of the targeted synovitis. Despite the use of higher volumes of permanent embolics (median 4.5 mL per intervention) and treatment of a greater number of vessels (median four per intervention) than reported in previous studies, reperfusion of previously treated territories occurred [20, 25]. The chronic synovitis underlying OA pain therefore represents a dynamic state that GAE aims to shift toward a steady state. In this context, reperfusion may be regarded as a hallmark of insufficient control and inflammatory progression. However, to maintain a steady state, GAE treatment strategies must be adapted to the extent of inflammation while accounting for the inherent technical limitations of the procedure. Consequently, in patients with severe OA and often more pronounced inflammation, more aggressive treatment strategies may be required, including higher volumes of permanent embolics, less restrictive embolization endpoints when using temporary embolics or consideration of reGAE [26].

Despite reGAE, 12 patients failed to achieve clinically meaningful pain improvement after reintervention. Patient characteristics and interventional data did not differ significantly from responders, indicating an ongoing knowledge gap in patient selection.

This study has several limitations. The sample size was relatively small, which limited the feasibility of subgroup analyses. However, the study was designed with an exploratory character. The cohort included patients with severe OA and post-TKR pain, which may differ in underlying pain mechanisms and could limit comparability. However, in post-TKR cases, prosthesis failure and hemarthrosis were excluded prior to treatment. Thus, both therapeutically challenging groups were analyzed together, as a higher inflammatory burden was assumed in these patients, who were therefore considered primary candidates for a more intensive treatment strategy using reGAE.

Clinical outcome assessment was restricted to the NRS to minimize patient burden and facilitate follow-up compliance. Follow-up duration differed between groups, with 12 months available for initial GAE responders and 6 months for reGAE responders. Longer follow-up after repeat GAE is required to determine the durability of the observed clinical response. Imaging assessment relied on conventional radiography, which precluded evaluation of advanced analyses but was chosen to keep the study protocol pragmatic, as MRI is not routinely performed in all patients undergoing GAE.

ReGAE was performed only in initial non-responders for ethical reasons, resulting in an asymmetric paired structure. Although McNemar’s exact test was used to account for this distribution, the study design may limit full bidirectional comparability.

Quantitative angiographic parameters, including blush size and blush reduction ratio, are not yet validated and are influenced by technical factors such as DSA projection, injection pressure, and contrast dilution. In particular, variability in angiographic projection and catheter positioning between initial GAE and reGAE may reduce the comparability of blush measurements. Although DSA series were systematically screened and images with marked differences in projection or catheter position were excluded from analysis, subtle variations inherent to real-world procedural settings cannot be entirely eliminated. Angiography was performed under clinical routine conditions, in which the use of a standardized power injector is not routine. Contrast dilution was kept constant across all patients to ensure intra-study consistency. Variability in DSA acquisition length and angiographic phase may limit direct visual comparison of selected pre- and post-embolization frames. However, quantitative analyses were based on fusion of the complete DSA series to minimize phase-related bias. Blush segmentation was applied as an exploratory approach to quantify the otherwise subjective embolization endpoint and to increase objectivity.

In conclusion, this study demonstrates that reperfusion of previously treated vascular territories can occur in patients with severe OA and post-TKR despite the use of permanent embolic agents and may represent a potential mechanism of persistent pain after initial GAE. In this patient group, reGAE represents a safe second-line treatment associated with an increase in the proportion of responders and clinically significant pain relief. These findings further support the need to individualize embolization strategies and treatment intervals according to disease severity and patient-specific factors, as a one-size-fits-all approach appears unrealistic.

## Supplementary Information

Below is the link to the electronic supplementary material.

Supplementary file1 (DOCX 15 KB)

Supplementary file2 (DOCX 14 KB)

Supplementary file3 (DOCX 16 KB)

Supplementary file4 (DOCX 17 KB)
