# Supplementary material for: Repeated Genicular Artery Embolization Using Permanent Microspheres for Severe Osteoarthritis and Postsurgical Pain
Source: Cardiovasc Intervent Radiol. 2026 Mar 18;49(7):1370–81. doi: 10.1007/s00270-026-04410-w (PMC13337900; doi:10.1007/s00270-026-04410-w)
Supplement: Supplementary file 1 — Supplementary file1 (DOCX 15 KB) [file 270_2026_4410_MOESM1_ESM.docx]

**Supplement 1: Outcome after initial and repeat GAE**

Numeric rating scale (NRS) scores are presented as median (range) for the total cohort and stratified into initial GAE responders, repeat GAE responders, and non-responders. P-values refer to comparisons of baseline to each follow-up. Response was defined as achieving a minimal clinically important difference (MCID) of ≥ 2-point reduction in NRS compared to baseline

|  | **Total**  **(n=55)** | **GAE Responders**  **(n=23)** | **reGAE**  **Responders**  **(n=20)** | **Non-Responders**  **(n=12)** |
| --- | --- | --- | --- | --- |
| **Baseline**, median (range)  *(p-Value)* | 8 (3-10) | 8 (3-10) | 8 (3-10) | 6 (3-10) |
| **6 weeks**, median (range)  *(p-Value)* | 5 (1-10)  *(p < 0.0001)* | 5 (1-9)  *(p < 0.001)* | 6 (1-10)  *(p > 0.05)* | 4 (3-7)  *(p > 0.05)* |
| **3 months**, median (range)  *(p-Value)* | 5 (1-9)  *(p < 0.0001)* | 5 (1-9)  *(p < 0.01)* | 7 (3-9)  *(p > 0.05)* | 6 (2-8)  *(p > 0.05)* |
| **6 months**, median (range)  *(p-Value)* | 6 (1-10)  *(p < 0.01)* | 5 (1-8)  *(p < 0.001)* | 8 (4-10)  *(p > 0.05)* | 7 (3-10)  *(p > 0.05)* |
| **9 months**, median (range)  *(p-Value)* | 4 (1-10)  *(p < 0.0001)* | 4 (1-7)  *(p < 0.0001)* | 5 (1-8)  *(p < 0.0001)* | 6 (2-10)  *(p > 0.05)* |
| **12 months**, median (range)  *(p-Value)* | 5 (1-10)  *(p < 0.0001)* | 5 (1-8)  *(p < 0.001)* | 3 (1-7)  *(p < 0.0001)* | 6 (3-10)  *(p > 0.05)* |
