# Supplementary material for: Repeated Genicular Artery Embolization Using Permanent Microspheres for Severe Osteoarthritis and Postsurgical Pain
Source: Cardiovasc Intervent Radiol. 2026 Mar 18;49(7):1370–81. doi: 10.1007/s00270-026-04410-w (PMC13337900; doi:10.1007/s00270-026-04410-w)
Supplement: Supplementary file 2 — Supplementary file2 (DOCX 14 KB) [file 270_2026_4410_MOESM2_ESM.docx]

**Supplement 2: McNemar Table**

Responder status was compared using McNemar’s test for paired binary data, demonstrating a significant increase in the proportion of responders after repeated GAE (b = 20, c = 0, exact p < 0.001)

|  | **Repeated GAE responders (n)** | **Repeated GAE**  **non-responders (n)** |
| --- | --- | --- |
| **Initial GAE responders (n)** | 23 (a) | 0 (c) |
| **Initial GAE non-responders (n)** | 20 (b) | 12 (d) |
