# Supplementary material for: Repeated Genicular Artery Embolization Using Permanent Microspheres for Severe Osteoarthritis and Postsurgical Pain
Source: Cardiovasc Intervent Radiol. 2026 Mar 18;49(7):1370–81. doi: 10.1007/s00270-026-04410-w (PMC13337900; doi:10.1007/s00270-026-04410-w)
Supplement: Supplementary file 3 — Supplementary file3 (DOCX 16 KB) [file 270_2026_4410_MOESM3_ESM.docx]

**Supplement 3: Quantitative Blush Analyses in initial GAE responders**

Angiographic blush size before and after embolization and blush reduction ratio (BRR) are reported for all treated arteries and for individual genicular arteries in the subgroup of initial GAE responders. P-values indicate within procedure comparisons of pre- versus post-embolization blush size, reflecting the immediate embolization effect.

*Abbreviations: DGA: Descending Genicular Artery; SMGA: Superiomedial Genicular Artery; IMGA: Inferiomedial Genicular Artery; SLGA: Superolateral Genicular Artery; ILGA: Inferiolateral Genicular Artery; ARTA: Anterior Recurrent Tibial Artery.*

|  |  | **Initial GAE**  **(n=23)** | |
| --- | --- | --- | --- |
|  |  | Pre | Post |
| **All arteries** | Blush size, (mm^2^)  median (range) | 1586  (113-6621) | 102  (2-1387) |
|  | BRR,  median (range) | 0.93  (0.83-0.99) | |
|  | *p-Value* | *(p < 0.0001)* | |
| **DGA** | Blush size, (mm^2^)  median (range) | 1388  (384-4458) | 150  (10-698) |
|  | BRR,  median (range) | 0.93  (0.80-0.99) | |
|  | *p-Value* | *(p < 0.0001)* | |
| **SMGA** | Blush size, (mm^2^)  median (range) | 1096  (410-3500) | 247  (114-314) |
|  | BRR,  median (range) | 0.80  (0.64-0.91) | |
|  | *p-Value* | *(p < 0.001)* | |
| **IMGA** | Blush size, (mm^2^)  median (range) | 1654  (113-4303) | 166  (4-950) |
|  | BRR,  median (range) | 0.89  (0.78-0.99) | |
|  | *p-Value* | *(p < 0.01)* | |
| **SLGA** | Blush size, (mm^2^)  median (range) | 1840  (183-6111) | 225  (17-899) |
|  | BRR,  median (range) | 0.89  (0.78-0.99) | |
|  | *p-Value* | *(p < 0.01)* | |
| **ILGA** | Blush size, (mm^2^)  median (range) | 1699  (117-3554) | 423  (6-1753) |
|  | BRR,  median (range) | 0.86  (0.74-0.99) | |
|  | *p-Value* | *(p < 0.05)* | |
| **ARTA** | Blush size, (mm^2^)  median (range) | 1489  (280-4783) | 115  (76-243) |
|  | BRR,  median (range) | 0.94  (0.83-0.99) | |
|  | *p-Value* | *(p > 0.05)* | |
