# Supplementary material for: Repeated Genicular Artery Embolization Using Permanent Microspheres for Severe Osteoarthritis and Postsurgical Pain
Source: Cardiovasc Intervent Radiol. 2026 Mar 18;49(7):1370–81. doi: 10.1007/s00270-026-04410-w (PMC13337900; doi:10.1007/s00270-026-04410-w)
Supplement: Supplementary file 4 — Supplementary file4 (DOCX 17 KB) [file 270_2026_4410_MOESM4_ESM.docx]

**Supplement 4: Quantitative Blush Analyses in repeat GAE responders and non-responders**

Angiographic blush size before and after embolization and blush reduction ratio (BRR) are reported for all treated arteries and for individual genicular arteries in the subgroup of repeated GAE responders (n=20) and non-responders (n=12). For initial GAE and repeat GAE (reGAE), p-values indicate within procedure comparisons of pre- versus post-embolization blush size, reflecting the immediate embolization effect. Additional p-values compare post-embolization blush size after initial GAE with pre-embolization blush size before reGAE, demonstrating reperfusion of previously treated vascular territories.

*Abbreviations: DGA: Descending Genicular Artery; SMGA: Superiomedial Genicular Artery; IMGA: Inferiomedial Genicular Artery; SLGA: Superolateral Genicular Artery; ILGA: Inferiolateral Genicular Artery; ARTA: Anterior Recurrent Tibial Artery.*

|  |  | **Initial GAE**  **(n=32)** | | **Repeat GAE**  **(n=32)** | |
| --- | --- | --- | --- | --- | --- |
|  |  | Pre | Post | Pre | Post |
| **All arteries** | Blush size, (mm^2^)  median (range) | 1607  (108-8057) | 102  (2-1753) | 1082  (234-9332) | 73  (3-1400) |
|  | BRR,  median (range) | 0.93  (0.80-0.99) | | 0.93  (0.85-0.99) | |
|  | *p-Value* | *(p < 0.0001) (p < 0.0001) (p < 0.0001)* | | | |
| **DGA** | Blush size, (mm^2^)  median (range) | 2195  (108-7719) | 107  (2-1409) | 934  (234-2691) | 52  (3-845) |
|  | BRR,  median (range) | 0.93  (0.84-0.99) | | 0.91  (0.75-0.99) | |
|  | *p-Value* | *(p < 0.0001) (p < 0.001) (p < 0.0001)* | | | |
| **SMGA** | Blush size, (mm^2^)  median (range) | 1421  (446-3850) | 60  (4-342) | 1037  (368-9294) | 48  (4-1400) |
|  | BRR,  median (range) | 0.94  (0.77-0.99) | | 0.95  (0.88-0.99) | |
|  | *p-Value* | *(p < 0.001) (p < 0.0001) (p < 0.0001)* | | | |
| **IMGA** | Blush size, (mm^2^)  median (range) | 1741  (128-6971) | 142  (3-627) | 1043  (282-9332) | 68  (8-403) |
|  | BRR,  median (range) | 0.94  (0.80-0.99) | | 0.93  (0.89-0.99) | |
|  | *p-Value* | *(p < 0.0001) (p < 0.0001) (p < 0.0001)* | | | |
| **SLGA** | Blush size, (mm^2^)  median (range) | 1461  (294-7926) | 113  (7-633) | 1230  (200-7840) | 60  (2-664) |
|  | BRR,  median (range) | 0.91  (0.70-0.99) | | 0.91  (0.79-0.99) | |
|  | *p-Value* | *(p < 0.01) (p < 0.001) (p < 0.0001)* | | | |
| **ILGA** | Blush size, (mm^2^)  median (range) | 2396  (246-8057) | 53  (3-1753) | 1208  (474-6703) | 118  (19-837) |
|  | BRR,  median (range) | 0.92  (0.75-0.99) | | 0.96  (0.82-0.99) | |
|  | *p-Value* | *(p < 0.0001) (p < 0.0001) (p < 0.0001)* | | | |
| **ARTA** | Blush size, (mm^2^)  median (range) | 971  (870-2103) | 58  (30-77) | 659  (348-971) | 26  (22-30) |
|  | BRR,  median (range) | 0.95  (0.92-0.99) | | 0.87  (0.86-0.87) | |
|  | *p-Value* | *(p > 0.05) (p > 0.05) (p > 0.05)* | | | |
